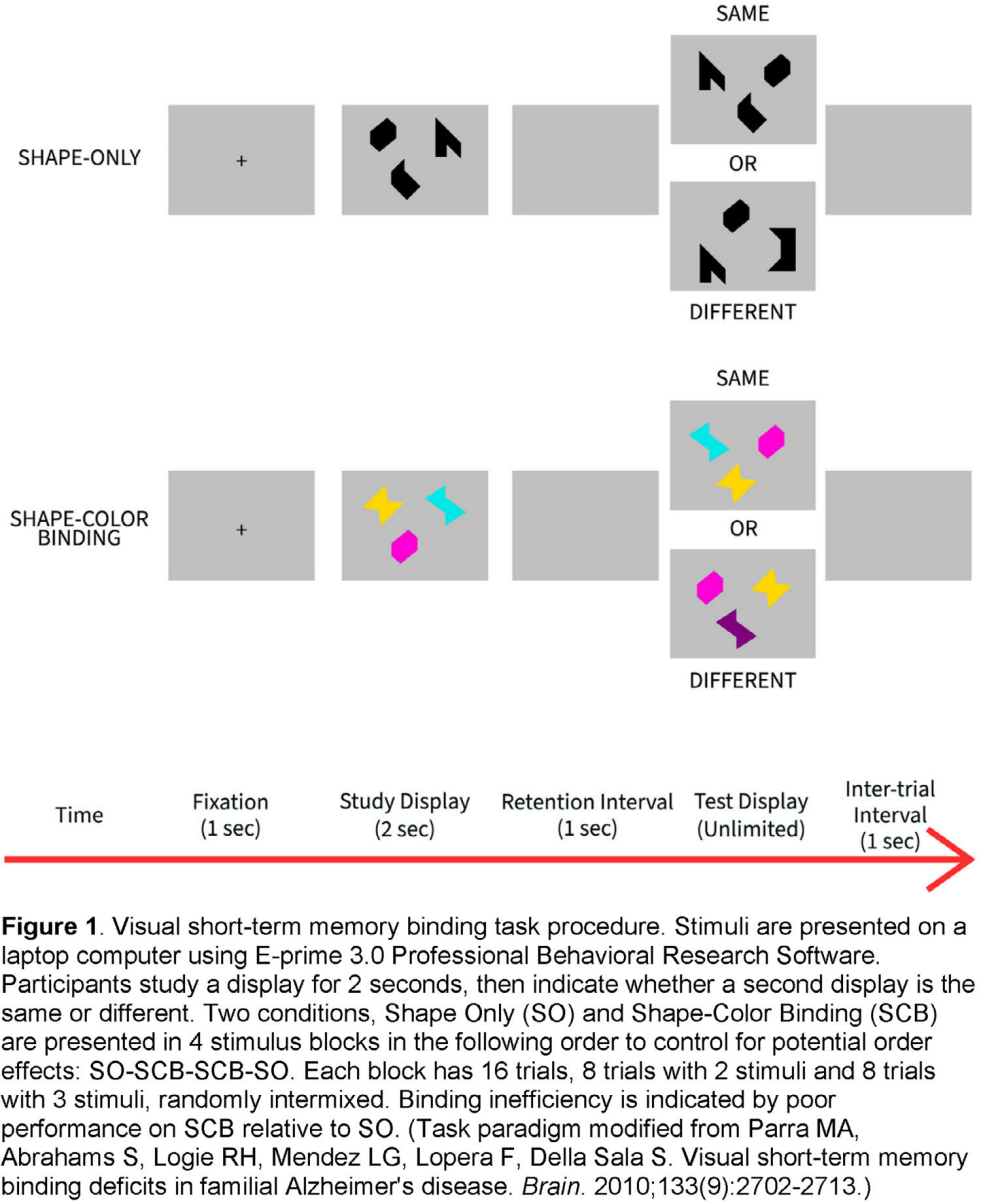# Visual short‐term memory binding, locus coeruleus integrity, and subjective cognitive decline in cognitively unimpaired older adults

**DOI:** 10.1002/alz70857_107146

**Published:** 2025-12-26

**Authors:** Diane M. Jacobs, Tyler R. Bell, Nathan Whitsel, Matthew S. Panizzon, Jeremy A. Elman, Chandra A. Reynolds, Christine Fennema‐Notestine, Eric L. Granholm, Amy J. Jak, Rosemary Toomey, Anders M. Dale, Michael J. Lyons, Carol E. Franz, William S. Kremen, for the Vietnam Era Twin Study of Aging

**Affiliations:** ^1^ Department of Neurosciences, University of California San Diego, La Jolla, CA, USA; ^2^ UCSD Shiley‐Marcos Alzheimer's Disease Research Center, La Jolla, CA, USA; ^3^ Center for Behavior Genetics of Aging, University of California, San Diego, La Jolla, CA, USA; ^4^ University of California San Diego, La Jolla, CA, USA; ^5^ University of California, San Diego, La Jolla, CA, USA; ^6^ Institute for Behavioral Genetics, University of Colorado Boulder, Boulder, CO, USA; ^7^ University of California, San Diego, San Diego, CA, USA; ^8^ Boston University, Boston, MA, USA; ^9^ Center for Multimodal Imaging and Genetics, University of California, San Diego, La Jolla, CA, USA

## Abstract

**Background:**

Visual short‐term memory binding (VSTMB) requires efficient functional connectivity between cortical regions and is a sensitive behavioral marker of Alzheimer's disease (AD). VSTMB impairments have been detected in individuals with subjective cognitive decline (SCD) who perform normally on standard neuropsychological tests. Research has linked SCD to reduced integrity of the rostral‐middle locus coeruleus (LC), an area that accumulates tau in early preclinical AD. Since the LC plays a crucial role in maintaining cortical efficiency, VSTMB may be similarly associated with LC integrity, particularly among those with SCD.

**Method:**

Data were from cognitively unimpaired men in the Vietnam Era Twin Study of Aging (*N* = 350; mean age=72.9, SD=2.4) who completed a test of VSTMB, an LC‐sensitive MRI scan, and the 39‐item Everyday Cognition (ECog) scale; 274 participants also had informant ECog ratings. The VSTMB task employs a change detection paradigm and compares performance in shape‐color binding (SCB) versus shape‐only (SO) conditions (see Figure 1). Rostral‐middle and caudal LC integrity was calculated as a contrast‐to‐noise ratio (LC‐CNR) using a pontine tegmentum reference region. Mixed models regressed VSTMB accuracy across condition (SO, SCB) and set size (2, 3) within person, while testing main effects and interactions with LC‐CNR and ECog. Models adjusted for age 20 cognitive ability, current age, depressive symptoms, and state anxiety. Sensitivity analyses adjusted for global performance on standard neuropsychological measures.

**Result:**

VSTMB accuracy was lower on the SCB than SO condition (b=‐1.08, *p* = .001), especially at higher set sizes (b=‐1.90, *p* < .001). Higher participant‐rated, but not informant‐rated, SCD was associated with decreasing accuracy on SCB relative to the SO condition (b=‐.253, *p* = .007). Lower rostral‐middle, but not caudal, LC‐CNR was associated with poorer SCB accuracy relative to SO condition accuracy (b=7.10, *p* = .010). Results remained significant after adjusting for global neuropsychological performance.

**Conclusion:**

Subtle losses in cognitive efficiency detected in cognitively unimpaired older adults on a VSTMB task were associated with SCD and LC integrity, even after accounting for performance on traditional neuropsychological tests. Given its role in modulating cortical efficiency through cognitive effort, reduced LC integrity may be associated with SCD when capacity for increasing compensatory effort to perform tasks is exceeded.